# How land-use intensity affects sexual and parthenogenetic oribatid mites in temperate forests and grasslands in Germany

**DOI:** 10.1007/s10493-020-00586-z

**Published:** 2021-02-09

**Authors:** Katja Wehner, Romina Schuster, Nadja K. Simons, Roy A. Norton, Nico Blüthgen, Michael Heethoff

**Affiliations:** 1 Ecological Networks, Technische Universität Darmstadt, Schnittspahnstraße 3, 64287 Darmstadt, Germany; 2 Institut für Bodenkunde und Standortlehre, Universität Hohenheim, Emil-Wolff-Straße 27, 70599 Stuttgart, Germany; 3 College of Environmental Science and Forestry, State University of New York, 1 Forestry 16 Drive, Syracuse, NY 13210 USA

**Keywords:** Biodiversity Exploratories, Land-use factors, Oribatid mites, Microarthropods, Sexuality, Parthenogenesis

## Abstract

Intensive land use has been shown to alter the composition and functioning of soil communities. Due to their low dispersal ability, oribatid mites are particularly vulnerable to land-use intensification and species which are not adjusted to management-related disturbances become less abundant. We investigated how different land-use parameters in forests and grasslands affect oribatid mite diversity and abundance, with a focus on: (1) species-level impacts, by classifying species as increasing (‘winners’) or decreasing (‘losers’) in abundance with higher land-use intensity, and (2) reproductive impact, by investigating whether sexual and parthenogenetic species react differently. We collected 32,542 adult oribatid mites in 60 forests and grasslands of known land-use intensity in two regions of Germany. Diversity and total abundance as well as the proportion of sexual species were higher in forests than in grasslands. Diversity declined with higher land-use intensity in forests, but increased with higher mowing and fertilization in grasslands. Depending on land-use parameter and region, abundance either declined or remained unaffected by increasing intensity. Gravidity was higher in sexual than in parthenogenetic species and sexuals had 1.6× more eggs per gravid female. Proportions of sexual species and gravid females decreased with land-use intensity in forests, but increased with mowing in grasslands. At the species level, 75% of sexuals and 87.5% of parthenogens were ‘losers’ of higher percentages of dead wood originating from management-related disturbances. Across land-use parameters and habitats, a similar proportion of sexual and parthenogenetic oribatid mite species were ‘losers’ of high land-use intensity. However, ‘winner’ species were more common among sexuals.

**Supplementary information:**

The online version of this article (10.1007/s10493-020-00586-z) contains supplementary material, which is available to authorized users.

## Introduction

Land-use intensification in forest and grassland habitats is often negatively correlated with the abundance and diversity of plant and animal taxa due to substantial changes in the environment (e.g., Altwood et al. 2008; Birkhofer et al. 2008; Culman et al. 2010; Allan et al. 2015). In agricultural systems, intensive mowing, grazing or fertilization causes the loss of soil organic matter and reduces the ability of soils to retain nutrients (Liiri et al. 2012; Birkhofer et al. 2012). In forests, intensive tree harvesting or the cultivation of non-native trees for the wood industry changes tree composition and influences the availability of resources in the soil food web system (Klarner et al. 2014). As these changes have a negative impact on biodiversity, the local and global impact of land-use intensity in forests and grasslands has increasingly been studied over recent decades (e.g., Marshall 2000; Allan et al. 2015; Birkhofer et al. 2012, 2017; Gossner et al. 2016; Penone et al. 2019).

The type and intensity of land-use vary in space and time and the associated degradation, fragmentation and conversion of habitats affect biota at different trophic levels (Newbold et al. 2005; Birkhofer et al. 2017). Arthropod richness, for instance, decreases with higher land-use intensity in agricultural ecosystems (Attwood et al. 2008; Culman et al. 2010; Chisté et al. 2016) and in highly managed forests (Zaitsev et al. 2002). Depending on the arthropod group, up to 50% of all species in a community were significantly less abundant under conditions of high land-use intensity (called ‘losers’; Chisté et al. 2016, 2018; Mangels et al. 2017).

Microarthropods of soil and litter are important for ecosystem processes (e.g., Seastedt and Crossley 1981; Wallwork 1983; Norton 1990). Oribatid mites are among the smallest groups (about 100–1000 μm body length) inhabiting almost all terrestrial microhabitats around the world in high diversity and density (Schatz and Behan-Pelletier 2008; Maraun and Scheu 2000; Cianciolo and Norton 2006; Schatz et al. 2011). They are substantially involved in nutrient dynamics, play an important role in decomposition and affect porosity of soils, aeration, infiltration and the distribution of organic matter within soils (Bird et al. 2004). Oribatid mites interact with the microbial soil community which promotes nutrient availability and plant growth (Seastedt 1984; Marshall 2000). Oribatid mites have species-specific dispersal rates between 0.3 and 2.1 cm per day (Lehmitz et al. 2012) and several species cannot easily escape from unfavorable conditions (Behan-Pelletier 1999; Lindo and Visser 2004) and often show great decrease in abundances following disturbances (Seastedt and Crossley 1981).

A special trait of oribatid mites is the high percentage (about 10%) of known or suspected parthenogenetic species (Norton and Palmer 1991). Theories predict that under certain environmental conditions sexual species have advantages over parthenogens due to the production of genetically diverse offspring (Maynard Smith 1978; Bell 1982; Lehtonen et al. 2012). On the other hand, advantages of parthenogenesis include a high reproductive potential due to the production of all-female offspring, no predation risk while mating and the maintenance of favorable gene combinations (Maynard Smith 1978; Bell 1982). However, whereas sex prevails in spatially heterogeneous habitats with diverse niches and structured resources in short supply, its advantage vanishes in the absence of resource limitation (Scheu and Drossel 2007; Maraun et al. 2019). The coexistence of sexual and parthenogenetic oribatid mite species in the same habitat allows the investigation if species with different reproductive modes are differently affected by increasing land-use intensity. When regular disturbances of their natural environment force oribatid mite species to quickly adapt to changing conditions, this may be realized by either producing genetically diverse offspring (sexuals) or by producing all-female populations to quickly (re-)colonize new or disturbed habitats (parthenogens; Lehmitz et al. 2012). The comparison of the current coexistence pattern among different land-use intensities can elucidate which reproductive mode performs better under certain conditions.

Belowground communities are proposed to be less sensitive to land-use intensity as the soil system buffers disturbances (Allan et al. 2015; Wolters 2001). Other studies found that mite community composition is affected by forest type (beech forests of different ages and coniferous stands; Erdmann et al. 2012; Maraun et al. 2012; Ehnes et al. 2014; Bluhm et al. 2016). In addition, effects of abiotic conditions such as soil pH, carbon of soil and fine root content have also been shown (Birkhofer et al. 2012; Erdmann et al. 2012). However, comparisons of land-use effects among microhabitats (grassland vs. forests) are less frequent and studies have focused on general community measures, such as abundance and diversity. Few studies have looked at functional composition or different life-history strategies so far (but see Birkhofer et al. 2016, 2017).

In the present study, we briefly characterized oribatid mite communities in forest and grassland habitats in two regions of Germany (the Swabian Alb in the South and the Schorfheide-Chorin in the North-East). We focused on (1) what impact of land-use intensity can be seen on the species-level of oribatid mites, and (2) whether sexual and parthenogenetic oribatid mite species are differently affected. On the species level, we classified species as ‘winners’ if they significantly increase in abundance and occurrence with land-use intensity, whereas ‘losers’ significantly decrease (compared to a statistical null model). For comparing sexual and parthenogenetic oribatid mites, we determine the proportion of females within species, their proportion of gravidity and the numbers of eggs within gravid females as a measure of reproductive success. We tested whether the proportion of gravidity and numbers of eggs are affected by land use in grassland and forests. We further investigated whether sexual or parthenogenetic species can more easily cope with changing environmental conditions due to land-use management.

## Material and methods

### Sampling sites

Oribatid mites were collected from established forest and grassland plots in two regions of Germany in 2017: the Swabian Alb and the Schorfheide-Chorin. These comprise research areas established in the framework of the Biodiversity Exploratory project (http://www.biodiversity-exploratories.de; Fischer et al. 2010). The Swabian Alb is a low-mountain range in southwest Germany (460–860 m above sea level [a.s.l.], 09° 10′ 49″–09° 35′ 54″ E/48° 20′ 28″–48° 32′ 02″ N) having an annual precipitation of 800–930 mm and a mean temperature of 8–8.5 °C. The Schorfheide-Chorin (abbreviated Schorfheide) is a glacially formed landscape in northeast Germany (3–140 m a.s.l., 13° 23′ 27″–14° 08′ 53″ E/52° 47′ 25″–53° 13′ 26″ N), with an annual precipitation of 520–580 mm and a mean annual temperature of 6–7 °C.

Study plots are characterized by different intensities of land-use (Fischer et al. 2010; Blüthgen et al. 2012; Kahl and Bauhus 2014). In forests, plots are 100 × 100 m in size. Parameters chosen to describe land-use comprise the proportion of harvested trees (Iharv), the proportion of non-native trees (Inonat) and the proportion of dead wood showing saw cuts (Idwcut). Iharv describes the proportion of harvested tree volume within a stand and is estimated by the presence of cut stumps and calculated as the ratio of harvested volume to the sum of standing, harvested and dead wood volume (Kahl and Bauhus 2014). Inonat is estimated as the proportion of harvested, living and dead wood volume of non-natural tree species to the sum volume of all tree species. Idwcut represents the proportion of dead wood with saw cuts to the total amount of dead wood (Kahl and Bauhus 2014). These three parameters are combined in a forest management index (Formi) and equally weighted (Kahl and Bauhus 2014). Formi, Iharv, Inonat and Idwcut were obtained from the Bexis database for the year 2016 (Online Appendix 1; ID 20055, owners: J. Hailer, U. Pommer, F. Van Broeck, M. Ayasse, V. Eisenbach).

In grasslands, plots are 50 × 50 m in size. Land-use parameters for grasslands comprise fertilization intensity (kg nitrogen ha^−1^ year^−1^), the mowing frequency per year and livestock grazing (livestock units*days of grazing ha^−1^ year^−1^), all combined in the land-use index LUI (Blüthgen et al. 2012). Data were obtained from the Bexis database and averaged for the years 2015 and 2016 (Online Appendix 1; ID 19266, owners: K. Lorenzen, W. Weisser, M. Ayasse, M. Fischer, J. Vogt).

### Sampling method

In 2017, soil samples were taken from a total of 60 plots in the Swabian Alb and the Schorfheide: 15 forest and 15 grassland plots in each of the regions. Plots, which were at least 5 km apart, were chosen according to their land-use intensity (Formi in forests and LUI in grasslands) to cover a complete gradient from low to high intensity (Online Appendix 1). Sampling was replicated 5 × per plot, at the south-east, south-west and north-west corners and in the middle of the two included edges, resulting in 300 samples in total. Each soil sample (20 × 20 cm in grasslands, 15 × 15 cm in forests, all 3 cm deep) was cut from the substrate using a sharp knife and included the surface materials, such as herbaceous vegetation, dead litter, mosses and pieces of wood.

Samples were directly transferred to the laboratory and oribatid mites were extracted by heat (increasing from 20 to 50 °C with 2 °C h^–1^) using a modified Kempson extractor for 48 h and collected into 75% ethanol (Kempson et al. 1963). Adult individuals were identified to species level whenever possible (not done for most Brachychthoniidae, Phthiracaridae and Suctobelbidae) using the identification key of Weigmann (2006). Sex was determined and, for females, the eggs were counted. Reproductive mode—sexual or parthenogenetic—was assigned to each species based on the sex ratio (assumed sexual if at least 20% of collected mites were male) or inferred from the literature (Norton and Palmer 1991; Cianciolo and Norton 2006; Domes et al. 2007; Wehner et al. 2018). Furthermore, mean body size of species was inferred from Weigmann (2006). Data are deposited at the Bexis database ID 26446 and openly available under 10.25829/bexis.26446-3.

### Statistical analysis

#### Oribatid mite assemblages

All statistical analyses were performed with R v.3.5.2 (R Core Team 2018). We characterized oribatid mite assemblages by species diversity and abundance and compared habitats (forest vs. grassland) and regions (Swabian Alb vs. Schorfheide) using non-metric multidimensional scaling (NMDS). We used Jaccard as a dissimilarity index for the complete assemblage and sexual species but Horn-Morisita dissimilarity index for parthenogenetic species. NMDS was calculated with two dimensions (k = 2) using the function ‘metaMDS’ in the R package ‘vegan’ (Oksanen et al. 2019). We further calculated the community weighted mean (CWM) of oribatid mite body size per plot.

Numbers of collected individuals were standardized to abundances per m^2^ (Ind/m^2^) and number of species was used for calculating the effective Shannon diversity (e^H^; Jost 2006). Normal distribution and homogeneity of variances of residiuals were tested using the Shapiro and Levene test, respectively. Data were log(x + 0.1) transformed if necessary.

To compare oribatid mite assemblages among regions and habitats, Shannon diversity, density and the size CWM were analyzed as response variables, whereas region (Swabian Alb, Schorfheide) and habitat (forest, grassland) were fixed as explanatory variables using ANOVA followed by Tukey’s pairwise test. Plot was implemented as random factor.

#### Mode of reproduction, sex ratios and number of eggs

Proportions of sexual and parthenogenetic species and individuals, respectively, as well as the proportion of females per species (sex ratio) were analysed as response variables using generalized linear models (glm) with binomial distribution. Numbers of eggs per gravid female were analysed as response variable using glm with Poisson distribution. Plot was implemented as random factor.

#### Influence of land-use intensity

The effects of land use on diversity, abundance, size CWM, proportion of females, proportion of gravidity and number of eggs were tested for the combined land-use indices (Formi in forests, LUI in grasslands) as well as for their respective sub-parameters (Online Appendix 1).

Using linear regressions, we tested the response of mite diversity, mite abundance and CWM of body size to changes in the land-use parameters. The response of the proportion of sexual individuals, the proportion of females therein and the number of eggs in gravid females to changes in the land-use parameters were tested using general linear model with binomial and Poisson distribution, respectively.

Moreover, we calculated each species’ ‘environmental niche’ along the gradient of land-use intensity within each habitat. The niche optimum was calculated as the abundance-weighted mean (AWM) for species *i* as

$$ AWM_{i} = \sum\nolimits_{p = 1}^{15} {L_{p} } \cdot \frac{{a_{i,p} }}{{A_{i} }}, $$

where *L*_*p*_ is the land-use gradient value of plot *p*, *a*_*i,p*_ the abundance of species *i* in plot *p* and *A*_*i*_ the total abundance of species *i* across all 15 forest or 15 grasslands sites, respectively (Chisté et al. 2016).

As a proxy for the ‘niche breadth’ of each species, we also calculated the abundance-weighted standard deviation (AWSD) for each land-use index and sub-parameter analogously. To test whether AWMs and AWSDs statistically deviate from an expected random distribution, we compared the calculated values against the expected values obtained from a null model that distributes each species across *N*_*i*_ sites with the same probability, with *N*_*i*_ being the number of sites in which species *i* was found. The null model thus chooses values of the focal land-use parameter (LUI, Formi, single components) of *N*_*i*_ sites and calculates a distribution of predicted AWMs and AWSDs values for each species based on 10,000 iterations. The null model was restricted to the one or two regions in which the species was recorded, to allow for potential distribution boundaries of each species in Germany that may not be related to plot conditions.

As in any randomization model, the percentage of AWMs or AWSDs from 10,000 null models with expected values greater or smaller than those observed, provides the *P*-value for the significance of the deviation between observed and expected values. A ‘winner’ is defined as a species with an observed AWM larger than the upper 5% of the distribution of AWMs obtained by the null models (i.e., associated with higher-than-average land-use intensity), a ‘loser’ shows an observed AWM smaller than the lower 5% (low land-use intensity specialist). For species that could be classified as neither ‘loser’ nor ‘winner’, we tested whether they are specialized on intermediate land-use or abiotic levels, that is, whether they have an intermediate AWM with a narrower niche than expected. Therefore, we compared the observed and expected weighted coefficient of variation (CV = AWSD/AWM) to account for the increase in standard deviation with increasing mean. This comparison allows us to distinguish ‘opportunists’ (observed CV ≥ expected CV) from species that are ‘specialized’ on intermediate land-use intensities (observed CV < expected CV and species not only occurring on one site, i.e., CV ≠ 0). As the definition of a weighted mean and weighted standard deviation is per se mathematically independent of the total abundance or total number of plots, rare species can be kept in the analysis.

## Results

### Habitat comparison

#### Oribatid mite assemblages

**Fig. 1:**
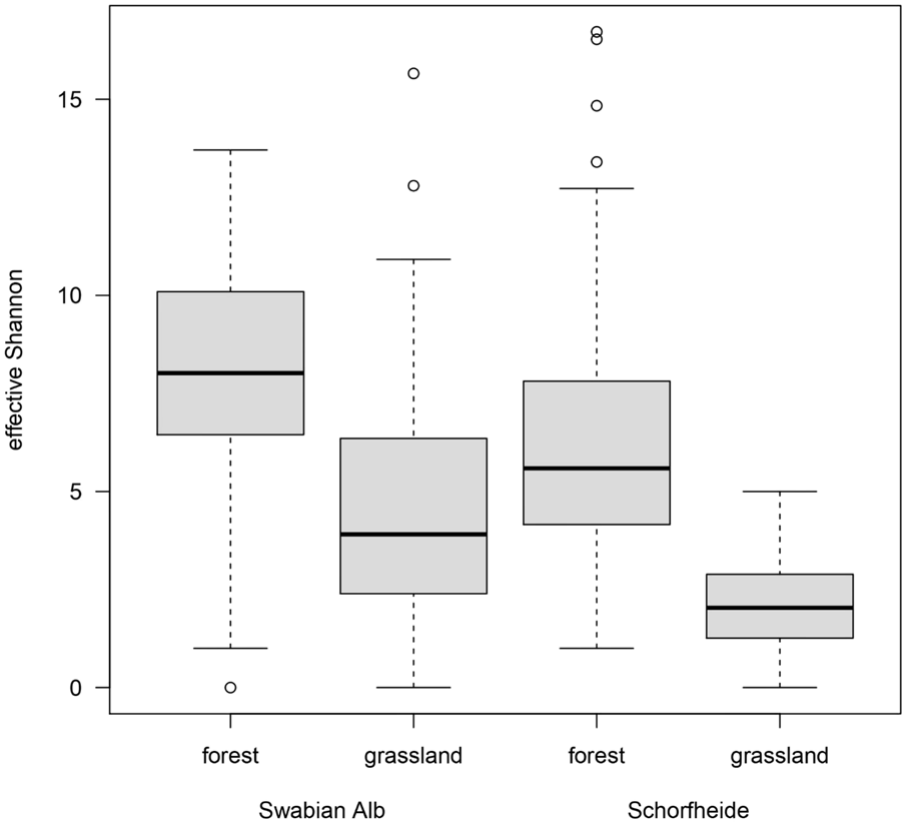
Effective Shannon diversity of oribatid mite communities in forest und grassland habitats in the Swabian Alb and the Schorfheide-Chorin. The black line within the boxplots represents the median of data, the upper and lower boxes the 75% and 25% quantile, respectively, and the whiskers 1.5× the interquartile range; outliers are represented by dots

In total, 17,611 adult oribatid mite individuals were collected in the Swabian Alb and 14,931 in the Schorfheide, belonging to 100 taxa in total (Table 1). In the Swabian Alb, 72 taxa were found in forests (15 parthenogenetic, 57 sexual) and 62 in grasslands (15 parthenogenetic, 47 sexual). Species richness was slightly lower in the Schorfheide; in forests, 70 taxa (25 parthenogenetic, 45 sexual) and in grasslands 34 taxa (8 parthenogenetic, 26 sexual) were represented. Similarly, oribatid mite diversity was consistently higher in forests than in grasslands albeit lower in the Schorfheide than in the Swabian Alb (Fig. 1; habitat: F_1,287_ = 124.839; region: F_1,287_ = 37.112, both p < 0.001).

**Table 1:** Species list, total abundance, percentage occurrence in forests and grasslands, percentage of females and eggs per gravid female of sexual and parthenogenetic oribatid mite species in the Swabian Alb and the Schorfheide-Chorin (numbers in *italics* summarize at the higher taxonomic levels)

Species	Sex	Swabian Alb							Schorfheide						
			Forest			Grassland				Forest			Grassland		
		Total # individuals	% Occurrence	% Females	Eggs per female	% Occurrence	% Females	Eggs per female	Total # individuals	% Occurrence	% Females	Eggs per female	% Occurrence	% Females	Eggs per female
Enarthronota		*150*	*41.56*	*100*	*1.0*	*58.44*	*100*	*1.0*	*156*	*98.60*	*100*	*1.0*	*1.40*	*100*	*1.0*
Brachychthoniidae	Parth	3	78.05	100	1.0	21.95	*–*	–	6	100	100	1.0	0	*–*	*–*
*Brachychthonius impressus*	Parth	0	–	–	–	–	–	–	4	100	100	1.0	0	*–*	*–*
*Liochthonius horridus*	Parth	0	–	–	–	–	–	–	2	100	100	1.0	0	*–*	*–*
*Poecilochthonius spiciger*	Parth	0	–	–	–	–	–	–	6	100	100	1.0	0	*–*	*–*
*Sellnickochthonius cricoides*	Parth	0	–	–	–	–	–	–	8	100	100	1.0	0	*–*	*–*
Eniochthoniidae															
*Eniochthonius minutissimus*	Parth	111	10.69	100	1.0	89.31	*100*	1.0	69	100	100	1.0	0	*100*	1.0
Hypochthoniidae															
*Hypochthonius rufulus*	Parth	36	98.42	100	*1.0*	1.58	*100*	1.0	63	97.07	100	*1.0*	2.93	*100*	1.0
Phthiracaroidea		*1079*	*96.35*	*–*	*5.5*	*3.65*	*–*	*3.4*	*264*	*100*	*–*	*4.7*	*0*	*–*	*–*
Phthiracaridae															
*Steganacarus* spp.	Sex	333	99.32	–	5.6	0.68	–	–	17	100	–	6.0	0	–	–
*Steganacarus boresetosus*	Sex	53	100	–	–	0	–	–	–	–	–	–	–	–	–
*Steganacarus carinatus*	Sex	50	100	–	–	0	–	–	–	–	–	–	–	–	–
*Steganacarus magnus*	Sex	76	98.50	–	9.4	1.50	–	–	33	100	–	11.0	0	–	–
*Steganacarus spinosus*	Sex	141	88.24	–	3.8	11.76	–	4.7	34	100	–	4.2	0	–	–
*Phthiracarus* spp.	Sex	380	97.28	–	5.2	2.72	–	3.0	48	100	–	6.0	0	–	–
*Phthiracarus boresetosus*	Sex	20	80.58	–	5.3	19.42	–	–	25	100	–	2.8	0	–	–
*Phthiracarus laevigatus*	Sex	13	100	–	7.0	0	–	–	71	100	–	4.8	0	–	–
Euphthiracaroidea															
Euphthiracaridae															
*Microtritia minima*	Parth	0	–	–	–	–	–	–	1	100	100	–	0	*–*	–
*Rhysotritia duplicata*	Parth	13	24.43	100	3.0	75.57	100	2.0	36	100	100	2.1	0	*–*	–
Desmonomata		*557*	*69.37*	*100*	*4.5*	*30.63*	*100*	*3.5*	*650*	*97.00*	*100*	*2.5*	*3.00*	*100*	*4.7*
Nothridae															
*Nothrus palustris*	Parth	19	90.46	100	3.0	9.54	100	1.5	7	100	100	4.0	0	*–*	*–*
*Nothrus parvus*	Parth	2	0	–	–	100	100	1.0	–	–	–	–	–	–	*–*
*Nothrus silvestris*	Parth	99	92.04	100	*2.0*	7.96	100	2.0	130	99.31	100	*2.0*	0.69	100	3.0
Camisidae															
*Platynothrus peltifer*	Parth	380	72.94	100	4.7	27.06	100	4.5	219	93.33	100	4.6	6.67	100	6.4
Nanhermanniidae															
*Nanhermannia nana*	Parth	57	19.93	100	1.3	80.07	100	1.2	294	98.59	100	1.9	1.41	100	1.7
Circumdehiscentiae		*15,825*	*72.31*	*67.9*	*1.7*	*27.69*	*68.2*	*2.1*	*13,861*	*88.24*	*78.0*	*1.6*	*11.76*	*69.6*	*2.6*
Hermanniidae															
*Hermannia gibba*	Sex	50	98.87	36.7	3.4	1.13	–	2.0	–	–	–	–	–	*–*	–
Damaeidae															
*Metabelba* sp.	Sex	17	27.59	66.7	4.0	72.41	58.3	3.2	369	99.54	35.7	2.9	0.46	*–*	–
*Damaeus* sp.	Sex	87	100	32.2	4.6	0	–	–	26	97.26	36.0	9.0	2.74	*–*	–
*Damaeus onustus*	Sex	25	100	11.1	4.0	0	–	–	–	–	–	–	–	*–*	–
*Damaeus riparius*	Sex	9	100	44.4	3.3	0	–	–	6	100	50.0	–	0	*–*	–
Cepheidae															
*Cepheus cepheiformis*	Sex	8	100	–	–	0	–	–	–	–	–	–	–	*–*	–
*Cepheus dentatus*	Sex	0	–	–	–	–	–	–	5	100	80.0	4.0	0	*–*	–
*Tritegeus bisculatus*	Sex	2	100	–	–	0	–	–	–	–	–	–	–	*–*	–
Damaeolidae															
*Fosseremus laciniatus*	Parth	23	14.48	–	1.5	85.52	–	1.0	–	–	–	–	–	*–*	–
Ctenobelbidae															
*Ctenobelba pectinigera*	Sex	30	16.49	–	–	83.51	71.4	3.0	–	–	–	–	–	–	–
Microzetidae															
*Microzetes septentrionalis*	Sex	13	100	61.5	1.3	0	–	–	–	–	–	–	–	–	–
Astegistidae															
*Cultroribula bicultrata*	Parth	22	97.39	100	1.2	2.61	–	–	21	94.12	100	1.8	5.88	100	2.0
Liacaridae															
*Adoristes ovatus*	Sex	3	100	33.3	–	0	–	–	3	100	50.0	–	0	–	–
*Liacarus subterraneus*	Sex	33	86.85	31.6	4.0	13.15	66.7	2.7	2	–	–	–	–	–	–
Peloppiidae															
*Ceratoppia* spp.	Sex	1	0	–	–	100	–	–	–	–	–	–	–	–	–
Carabodidae															
*Carabodes femoralis*	Sex	–	–	–	–	–	–	–	5	100	–	–	0	–	–
*Carabodes labyrinthicus*	Sex	104	48.48	57.1	3.1	51.52	66.7	3.0	4	100	–	–	0	–	–
*Carabodes ornatus*	Sex	61	93.20	81.8	4.1	6.80	50.0	4.0	78	99.12	57.4	3.5	0.88	–	–
*Carabodes reticulatus*	Sex	104	34.62			65.38			4	100			0	–	–
Tectocepheidae															
*Tectocepheus* spp.	Parth	1137	43.54	98.8	1.3	56.46	99.4	1.3	1359	73.90	99.5	1.1	26.10	100	1.2
Quadroppiidae															
*Quadroppia quadricarinata*	Parth	402	98.15	100	1.0	1.85	–	–	35	100	100	1.0	0	–	–
Oppiidae															
Oppiidae	Sex	–	–	–	–	–	–	–	8	100	62.5	–	0	–	–
*Berniella conjuncta*	Sex	–	–	–	–	–	–	–	62	100	58.6	1.0	0	–	–
*Berniella sigma*	Sex	46	7.48	–	–	92.52	44.7	1.0	18	100	72.2	–	0	*–*	–
*Dissorhina ornata*	Sex	580	98.73	59.5	1.4	1.27	53.8	1.3	1245	99.84	52.5	1.5	0.16	*–*	–
*Medioppia subpectinata*	Sex	2633	99.46	64.3	1.5	0.54	70.6	1.8	319	99.65	57.2	1.4	0.35	*–*	–
*Microppia minus*	Parth	33	19.69	100	1.0	80.31	100	1.0	15	100	100	–	0	*–*	–
*Multioppia laniseta*	Sex	–	–	–	–	–	–	–	1	100	–	–	0	*–*	–
*Oppiella falcata*	Sex	1026	88.86	53.5	1.0	11.14	45.1	1.0	479	100	59.8	1.3	0	*–*	–
*Oppiella nova*	Parth	1559	79.55	99.9	1.2	20.45	100	1.5	3266	98.90	100	1.3	1.10	100	1.1
Suctobelbidae															
*Allosuctobelba* spp.	Sex	8	100	–	–	0	–	–	–	–	–	–	–	*–*	–
*Suctobelbella* spp.	Parth	723	93.11	–	–	6.89	–	–	1534	99.91	–	1.0	0.09	*–*	–
*Suctobelbella acutidens*	Parth	–	–	–	–	–	–	–	340	100	–	1.0	0	*–*	–
*Suctobelbella forsslundi*	Parth	–	–	–	–	–	–	–	1	100	–	–	0	*–*	–
*Suctobelbella sarekensis*	Parth	–	–	–	–	–	–	–	88	100	–	1.0	0	*–*	–
*Suctobelbella similis*	Parth	–	–	–	–	–	–	–	45	100	–	–	0	*–*	–
*Suctobelbella subcornigera*	Parth	–	–	–	–	–	–	–	313	100	–	1.0	0	*–*	–
*Suctobelbella subtrigona*	Parth	1	100	–	–	0	–	–	204	100	–	1.0	0	*–*	–
Autognetidae															
*Conchogneta dalecarlica*	Sex	11	100	–	–	0	–	–	38	98.46	66.7	1.2	1.54	*–*	–
Thyrisomidae															
*Banksinoma lanceolata*	Sex	1	0	–	–	100	–	–	4	0	–	–	100	*–*	–
*Pantelozetes paolii*	Sex	167	90.21	58.9	1.4	9.79	56.6	1.2	75	89.14	57.4	1.2	10.86	90.9	1.0
Scutoverticidae															
*Scutovertex sculptus*	Sex	–	–	–	–	–	–	–	3	47.06	–	–	52.94	*–*	–
Phenopelopodae															
*Eupelops hirtus*	Sex	1	100	–	–	0	–	–	–	–	–	–	–	*–*	–
*Eupelops occultus*	Sex	28	57.14	66.7	2.0	42.86	33.3	3.3	58	67.95	61.5	3.5	32.05	64.3	3.1
*Eupelops plicatus*	Sex	453	10.13	56.5	4.0	89.87	47.8	3.3	48	42.99	69.2	3.0	57.01	60.7	3.3
*Eupelops tardus*	Sex	11	0	–	–	100	–	–	–	–	–	–	–	*–*	–
*Eupelops torulosus*	Sex	15	0	–	–	100	50.0	–	–	–	–	–	–	*–*	–
*Eupelops* spp.	Sex	1	100	–	–	0	–	–	6	84.21	–	–	15.79	*–*	–
*Peloptulus phaenotus*	Sex	1	0	–	–	100	–	–	6	100	–	–	0	*–*	–
Achipteriidae															
*Achipteria coleoptrata*	Sex	1032	93.71	56.6	2.7	6.29	64.5	2.4	587	92.69	55.5	2.4	7.31	58.7	2.4
*Achipteria nitens*	Sex	87	18.76	57.1	2.0	81.24	56.4	2.6	0	–	–	–	–	*–*	–
*Achipteria quadridentata*	Sex	7	100	–	–	0	–	–	56	98.68	72.2	2.0	1.32	–	–
Oribatellida															
*Ophidiotrichus tectorum*	Sex	33	96.50	46.7	1.0	3.50	–	–	6	100	20.0	–	0	*–*	–
*Ophidiotrichus vindobonensis*	Sex	–	–	–	–	–	–	–	1	100	–	–	0	*–*	–
*Oribatella calcarata*	Sex	36	98.42	37.5	4.0	1.58	–	–	–	–	–	–	–	*–*	–
*Oribatella quadricornuta*	Sex	24	95.14	52.9	4.5	4.86	–	–	36	94.67	41.7	3.3	5.33	*–*	–
Galumnidae															
*Galumna* sp.	Sex	110	52.34	64.3	1.5	47.66	63.9	3.1	63	96.07	40.0	2.0	3.93	*–*	–
*Galumna lanceata*	Sex	–	–	–	–	–	–	–	15	100	48.9	2.0	0	*–*	–
*Pergalumna nervosa*	Sex	–	–	–	–	–	–	–	26	100	50.0	4.0	0	*–*	–
Ceratozetidae															
*Ceratozetes peritus*	Sex	296	40.52	57.1	1.3	59.48	–	–	–	–	–	–	–	*–*	–
*Ceratozetes gracilis*	Sex	–	–	–	–	–	–	–	195	73.74	73.3	2.8	26.26	73.0	3.3
*Fuscozetes setosus*	Sex	57	92.70	51.4	2.3	7.30	83.3	1.3	–	–	–	–	–	–	–
*Lepidozetes singularis*	Sex	169	33.26	64.7	1.8	66.74	74.5	1.4	–	–	–	–	–	*–*	–
*Melanozetes meridianus*	Sex	19	96.97	–	–	3.03	–	–	–	–	–	–	–	–	–
*Trichoribates novus*	Sex	147	96.46	69.7	1.8	3.54	40.0	2.0	3	0	–	–	100	–	–
Chamobatidae															
*Chamobates borealis*	sex	260	85.56	46.8	2.8	14.44	44.5	6.2	–	–	–	–	–	*–*	–
*Chamobates cuspidatus*	Sex	626	98.45	50.0	5.5	1.55	47.1	2.0	527	96.07	45.7	5.1	3.93	40.0	2.4
*Chamobates subglobulus*	Sex	–	–	–	–	–	–	–	73	95.34	53.5	2.6	4.66	57.1	1.0
Punctoribatidae															
*Minunthozetes semirufus*	Sex	57	3.08	–	–	96.92	37.5	3.0	2	0	–	–	100	*–*	–
*Mycobates* sp.	Sex	7	70.33	–	–	29.67	–	–	–	–	–	–	–	*–*	–
*Puntoribates punctum*	Sex	1067	1.16	50.0	3.0	98.84	56.3	1.8	231	17.16	54.2	3.3	82.84	63.6	3.0
Euzetidae															
*Euzetes* sp.	Sex	17	96.60	80.0	1.0	3.40	–	–	–	–	–	–	–	–	–
Scheloribatidae															
*Liebstadia pannonica*	Sex	21	41.56	40.0	2.0	58.44	66.6	2.6	–	–	–	–	–	–	–
*Liebstadia similis*	Sex	807	0.66	66.7	1.0	99.34	56.3	1.9	717	1.49	44.0	2.8	98.51	60.6	2.1
*Scheloribates* spp.	Sex	–	–	–	–	–	–	–	3	0	–	–	100	–	–
*Scheloribates initialis*	Sex	309	32.56	56.6	3.8	67.44	64.4	3.3	70	100	52.4	2.3	0	–	–
*Scheloribates laevigatus*	Sex	909	6.09	64.3	3.8	93.91	62.9	3.1	970	46.65	55.2	2.7	53.35	58.5	3.0
Oribatulidae															
*Oribatula tibialis*	Sex	403	28.56	50.7	4.1	71.44	56.6	2.7	189	99.68	59.5	3.8	0.32	–	–
Oribatida		*17,611*							*14,931*						
Sexual individuals		13,005	74.24	60.4	2.3	74.48	58.6	2.5	6898	41.39	55.9	2.5	77.06	60.8	2.9
Parthenogenetic individuals		4506	25.76	99.7	1.9	25.52	99.8	1.8	8033	58.61	99.9	1.4	22.94	99.8	1.9

Italic numbers summarize higher taxonomic levels

**Fig. 2:**
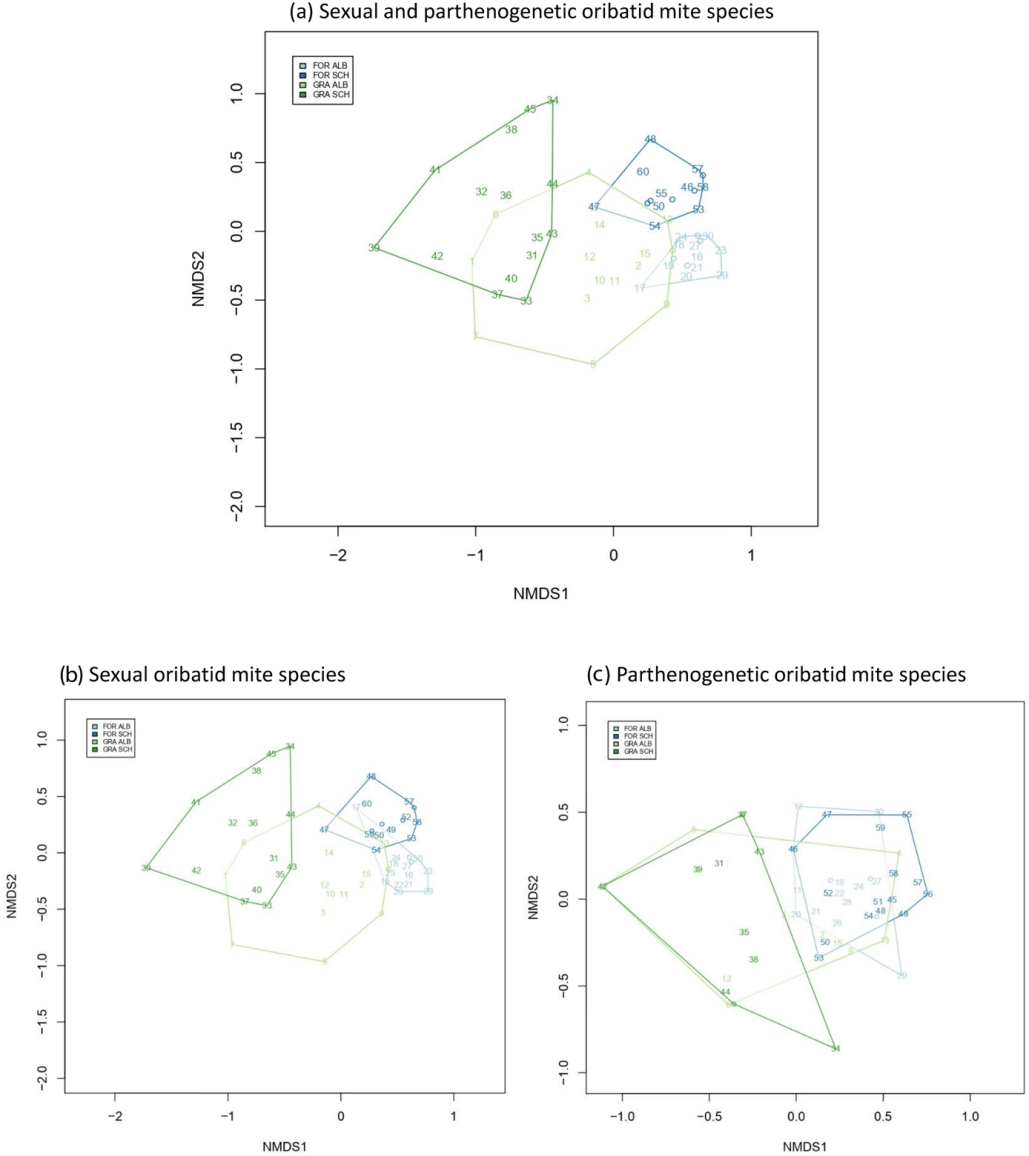
Non-metric multidimensional scaling (NMDS) for **a** sexual and parthenogenetic (Jaccard distance, k = 2), **b** sexual (Jaccard distance, k = 2) and **c** parthenogenetic (Horn distance, k = 2) oribatid mite assemblages in forests and grasslands in the Swabian-Alb and the Schorfheide-Chorin. Light blue: forest (FOR) in the Swabian Alb (ALB), blue: forest (FOR) in the Schorheide-Chorin (SCH), light green: grassland (GRA) in the Swabian Alb (ALB), green: grassland (GRA) in the Schorheide-Chorin (SCH)

**Fig. 3:**
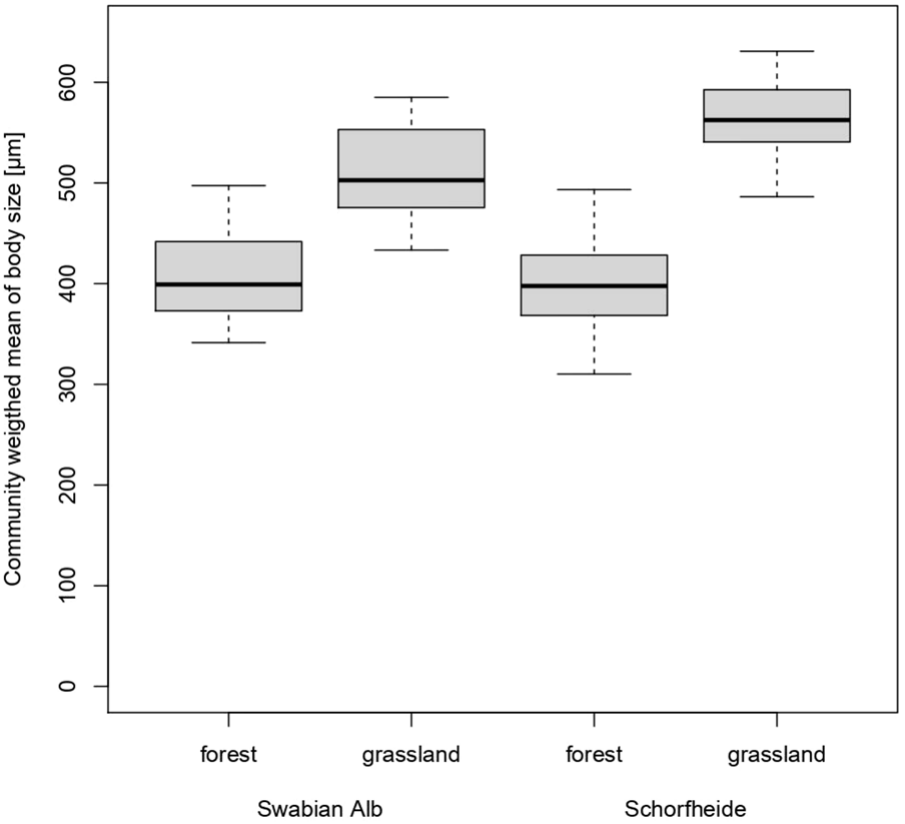
Community weighted mean of body size of oribatid mite assemblages in forest and grassland habitats in the Swabian Alb and the Schorfheide-Chorin. The black line within the boxplots represents the median of data, the upper and lower boxes the 75% and 25% quantile, respectively, and the whiskers 1.5× the interquartile range; outliers are represented by dots

NMDS analysis, including sexually and parthenogenetically reproducing oribatid mite species, clearly separated habitats and regions (Fig. 2a). However, assemblages were more similar to each other in the same habitat of different regions than among habitats in one region. NMDS analysis of sexual species revealed a similar pattern with a broader overlap of forest assemblages (Fig. 2b). In contrast, oribatid mite assemblages of parthenogenetic species were highly similar (Fig. 2c). The pattern of similar assemblages in similar habitats among regions is also present in the size CWM (Fig. 3; habitat: F_1,56_ = 113.838, p < 0.001; region: F_1,56_ = 3.268, p = 0.076). Assemblages in forests comprise smaller species than in grasslands which was more pronounced in the Schorfheide than in the Swabian Alb (habitat*region: F_1,56_ = 6.558, p = 0.013).

**Fig. 4:**
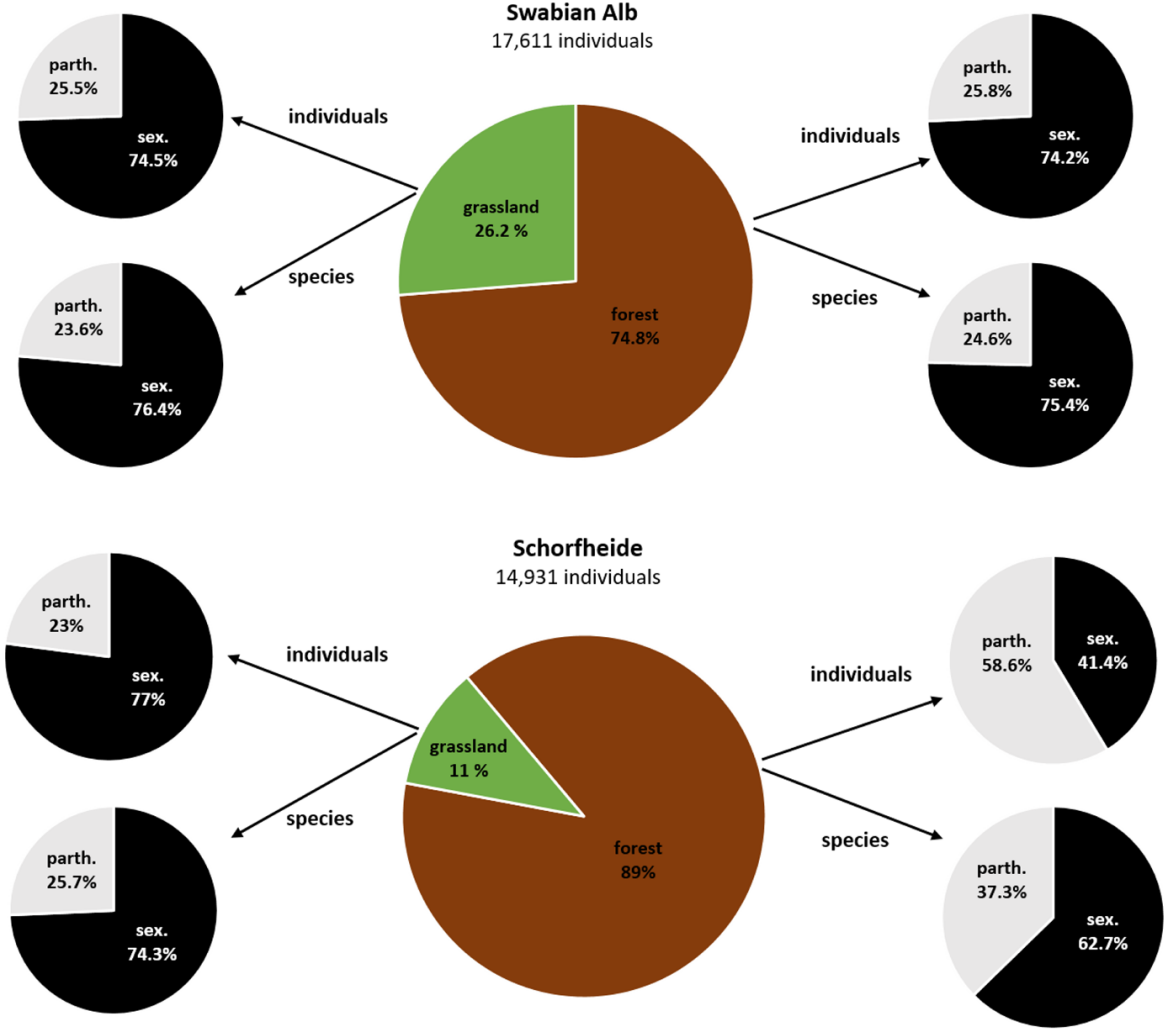
Percentage distribution of total oribatid mite individuals, sexual (sex.) and parthenogenetic (parth.) individuals and species in forest and grassland habitats in the Swabian Alb and the Schorfheide-Chorin

In both regions, the majority of adult oribatid mite individuals occurred in forests; this pattern was more pronounced in the Schorfheide (74.8 and 89%, respectively; Fig. 4 middle; habitat: F_1,287_ = 64.672, p < 0.001; region: F_1,287_ = 4.7, p = 0.031; habitat*region: F_1,287_ = 4.805, p = 0.029).

#### Mode of reproduction, sex ratio and number of eggs

The percentages of sexual and parthenogenetic individuals and species were similar in grassland and forest habitats in the Swabian Alb and in grasslands in the Schorfheide: with about 75%, sexuality dominated over parthenogenesis (Fig. 4 left and right). By contrast, Schorfheide forests had a higher percentage of parthenogenetic individuals (58.6%) and a relatively high percentage of parthenogenetic species (37.3%) (Table 2).

**Table 2:** Statistical analysis of the reproductive mode (sexual vs parthenogenetic) among habitats (forest vs grassland) and regions (Swabian Alb vs Schorfheide-Chorin)

	Df	denDf	F	p
Sex	1	69	4.604	0.035
Habitat	1	70	25.843	< 0.001
Region	1	70	0.036	0.85
Habitat:Sex	1	69	14.353	< 0.001
Region:Sex	1	69	5.660	0.020
Region:Habitat	1	70	5.317	0.024
Region:Habitat:Sex	1	69	6.865	0.011
Intercept	1	70	223.773	< 0.001

*Df* degrees of freedom, *den* denominator

**Fig. 5:**
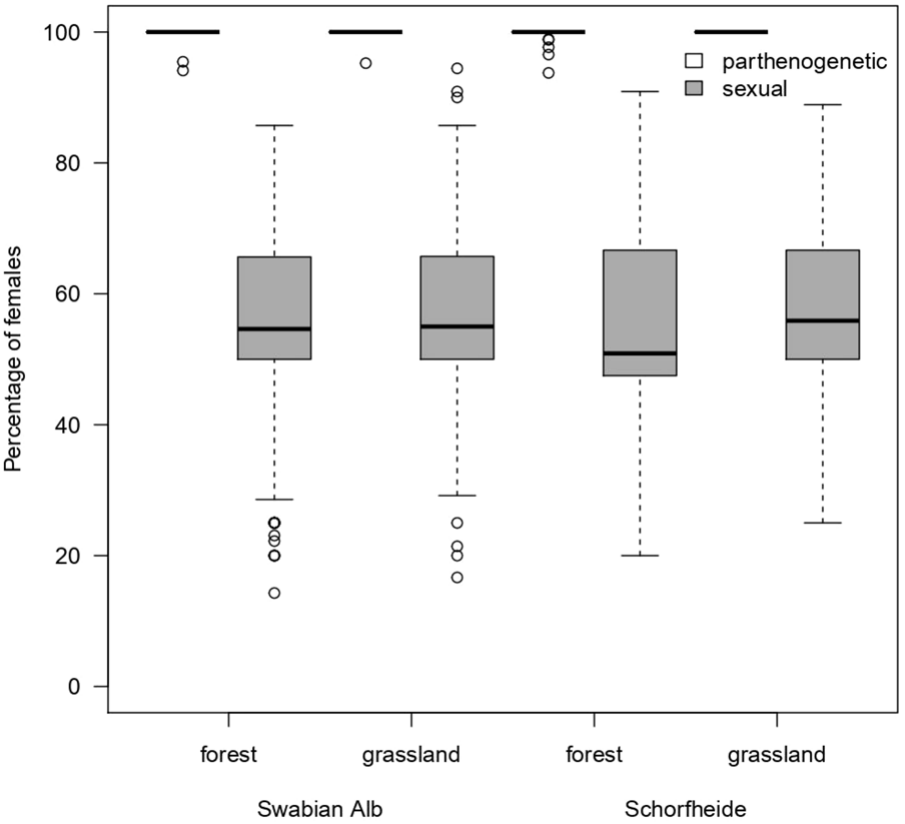
Percentage of females in sexual and parthenogenetic oribatid mite species in forest and grassland habitats in the Swabian Alb and the Schorfheide-Chorin. The black line within the boxplots represents the median of data, the upper and lower boxes the 75% and 25% quantile, respectively, and the whiskers 1.5× the interquartile range; outliers are represented by dots

The percentage of females in sexual and parthenogenetic oribatid mite communities significantly differed with the reproductive mode and region (Fig. 5, Table 3). In sexual species 55.3 and 54.9% females were present in the Swabian Alb and the Schorfheide, respectively. The parthenogenetic part of the oribatid mite community had almost 100% females in grasslands and had only a few males in forests (99.95% females).

**Table 3:** Statistical analysis (general linear model with binomial distribution) of the percentage of females within sexual and parthenogenetic oribatid mite species among forests and grasslands habitats in the Swabiab Alb and the Schorfheide-Chorin

	Df	Deviance	Resid. Df	Resid. Dev	F	p
Sex	1	540.87	2375	1128	1258.1	< 0.001
Habitat	1	0.98	2374	1127	2.288	0.13
Region	1	0.85	2373	1126.1	1.972	0.16
Sex:Habitat	1	1.45	2372	1124.7	3.369	0.067
Sex:Region	1	3.49	2371	1121.2	8.118	0.004
Habitat:Region	1	0.63	2370	1120.6	1.463	0.23
Sex:Habitat:Region	1	0.00	2369	1120.6	0.004	0.95
			2376	1668.8		

*Df* degrees of freedom, *Resid.* residuals, *Dev* deviation

The sex ratio of parthenogenetic and sexual species in forest and grassland communities was consistent among regions (Table 1). In sexual species, sex ratios varied between ca. 30% (*Ophidiotrichus tectorum*, *Oribatella calcarata*, *Hermannia gibba*, *Damaeus* spp.) and 70% females (*Ceratozetes gracilis*, *Achipteria quadridentata*; Table 1) and averaged at about 59%. Parthenogenetic species normally comprised 100% females, but rare, spanandric males were found in the genus *Tectocepheus* and in *Oppiella nova* (Table 1).

**Fig. 6:**
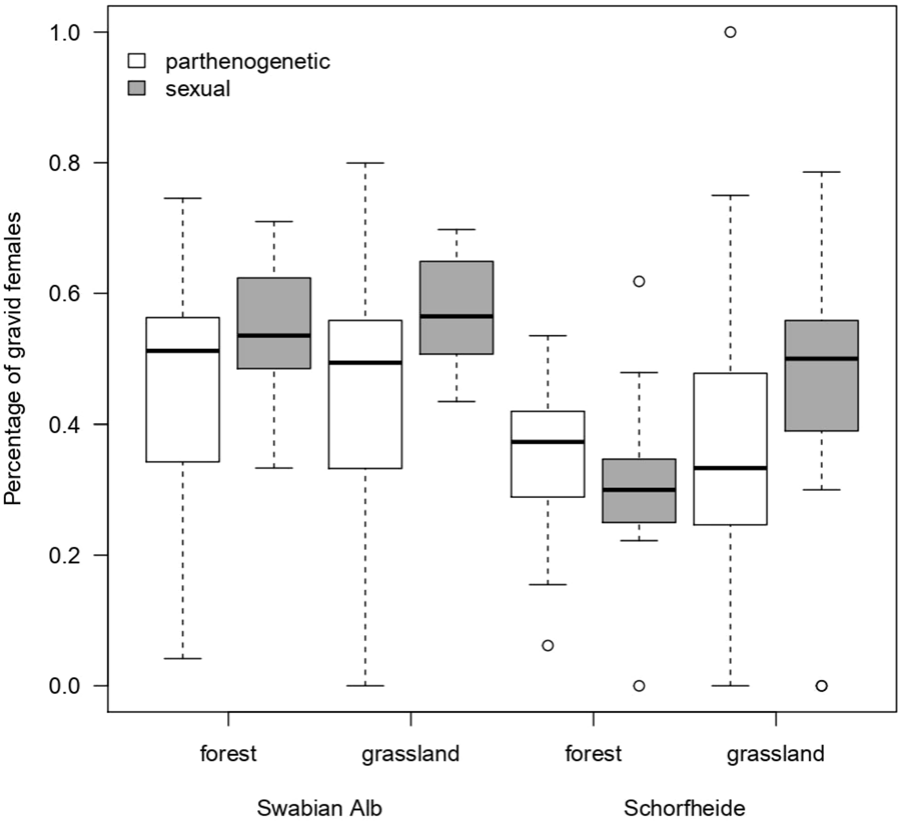
Percentage of gravid females in sexual and parthenogenetic oribatid mite species in forest and grassland habitats in the Swabian Alb and the Schorfheide-Chorin. The black line within the boxplots represents the median of data, the upper and lower boxes the 75% and 25% quantile, respectively, and the whiskers 1.5×  the interquartile range; outliers are represented by dots

**Fig. 7:**
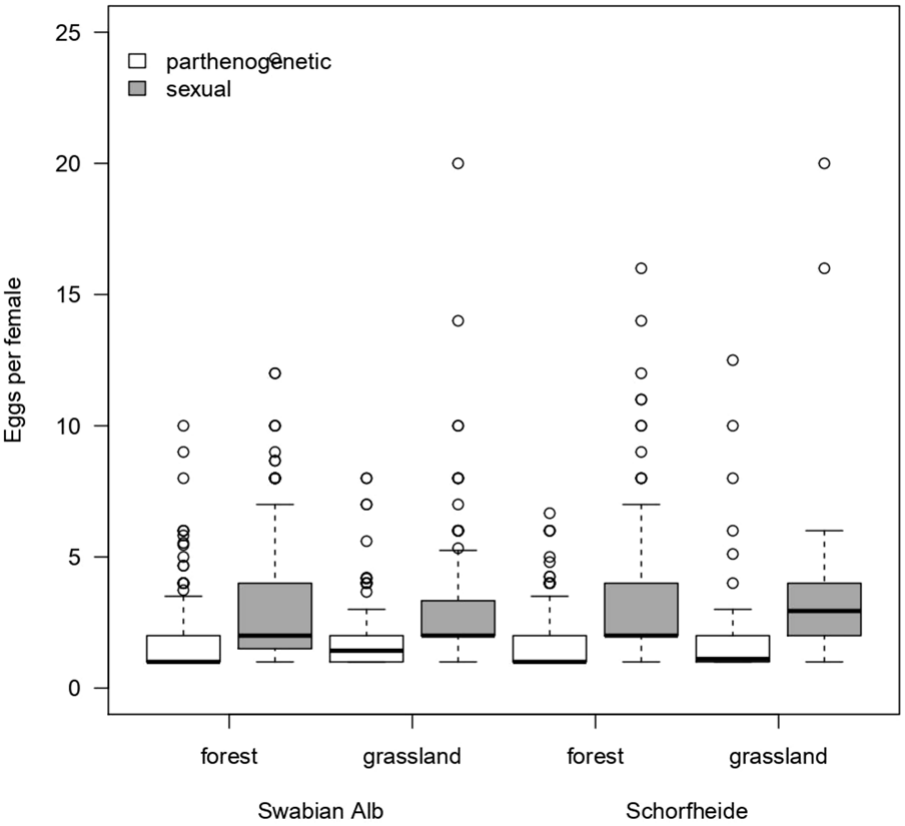
Numbers of eggs in gravid females in sexual and parthenogenetic oribatid mite species in forest and grassland habitats in the Swabian Alb and the Schorfheide-Chorin. The black line within the boxplots represents the median of data, the upper and lower boxes the 75% and 25% quantile, respectively, and the whiskers 1.5× the interquartile range; outliers are represented by dots

The average percentage of gravid female was generally higher in sexual than in parthenogenetic species (71 vs. 63%) and higher in the Swabian Alb than in the Schorfheide (72 vs. 62%; Fig. 6, Table 4). In both habitats and both regions, sexual species had significantly more eggs per gravid female than parthenogenetic species (2.97 vs. 1.81 on average; Fig. 7, Table 5).

**Table 4:** Statistical analysis (general linear model with binomial distribution and plot as random factor) of the percentage of gravid females among different habitats (forest vs grassland) and regions (Swabian Alb vs Schorfheide-Chorin)

	Df	denDf	F	p
Sex	1	69	4.759	0.033
Habitat	1	70	3.268	0.075
Region	1	70	22.755	< 0.001
Sex:Habitat	1	69	2.509	0.12
Sex:Region	1	69	1.223	0.27
Habitat:Region	1	70	1.855	0.18
Sex:Habitat:Region	1	69	0.713	0.40
Intercept	1	70	864.171	< 0.001

*Df* degrees of freedom, *den* denominator

**Table 5:** Statistical analysis (general linear model with poisson distribution) of eggs per gravid female in sexual and parthenogenetic oribatid mite species in forests and grasslands in the Swabian Alb and the Schorfheide-Chorin

	Df	Deviance	F	p
Sex	1	180.01	180.098	< 0.001
Habitat	1	0.092	0.092	0.76
Region	1	0.242	0.242	0.62
Sex: Habitat	1	4.926	4.926	0.026
Sex: Region	1	6.094	6.094	0.014
Habitat: Region	1	5.177	5.177	0.023
Sex: Habitat:Region	1	2.536	2.536	0.11

*Df* degrees of freedom

### Influence of land-use intensity

The community weighted mean (CWM) of body sizes was not affected by any land-use parameter neither in forests nor in grasslands (Table 6, for statistical values see Online Appendix 2). Diversity of oribatid mite assemblages correlated negatively with all forest-management parameters except wood harvesting in the Swabian Alb, and with the proportion of dead wood with saw cuts and the combined forest-management index in the Schorfheide (Table 6, Online Appendix 3). In grasslands, land-use intensity had a stronger impact on oribatid mite diversity than on density. In both regions, diversity was significantly lower on plots with a high land-use index and in the Swabian Alb additionally on plots with high grazing intensity. In contrast, diversity was correlated positively with high mowing and fertilization intensity in the Schorfheide (Table 6, Online Appendix 3).

**Table 6:** Influence of the land-use parameters forest management index (Formi), proportion of non-native trees (Inonat), proportion of dead wood with saw cuts (Idwcut) and the proportion of harvested trees (Iharv) in forests and the land-use index (LUI), grazing, mowing and fertilization in grasslands

	Forest		Grassland	
	Swabian Alb	Schorfheide	Swabian Alb	Schorfheide
Community weigthed mean of body size				
Diversity	Formi↓	Formi↓	LUI↓	LUI↓
	Inonat↓		Grazing↓	Mowing↑
	Idwcut↓	Idwcut↓		Fertilization↑
Density		Idwcut↓		Grazing↓
				Fertilization↓
Proportion of sexual individuals	Formi↓			Mowing↑
	Inonat↓			
Proportion of females in sexuals				LUI↓
				Mowing↑
				Grazing↓
Proportion of gravid females in sexuals	Inonat↓	Inonat↓	Fertilization↑	Fertilization↑
		Iharv↓		
		Idwcut↓		
Proportion of gravid females in parthenogens				Grazing↓
			Fertilization↑	Mowing↑
Eggs/female in sexuals				
Eggs/female in parthenogens	Iharv↓	Iharv↓		

Only signifcant effects are shown. ↓, negative effect; ↑, positive effect

In the Schorfheide, oribatid mite density significantly dropped with increasing proportion of dead wood with saw cuts in forests and with higher grazing and fertilization intensity in grasslands (Table 6, Online Appendix 3). However, density in the Swabian Alb was unaffected by either grassland or forest land-use parameters.

A high forest management index and a high number of non-native trees significantly correlated with lower proportion of sexual individuals in forests in the Swabian Alb, but mowing in grasslands was positively correlated with the proportion of sexual individuals in the Schorfheide (Table 6, Online Appendix 4). Accordingly, the proportion of females in sexual species was only affected in grasslands in the Schorfheide; the proportion of females was lower at high land-use indexes and high grazing intensity whereas intensive mowing was correlated positively with the proportion of females in sexual species.

In sexual species, the proportion of gravid females dropped with an increasing number of non-native trees, tree harvesting and dead wood with saw cuts; in grasslands, a higher proportion of gravid females was present on plots with high fertilization (Table 6, Online Appendix 5). In parthenogenetic species, gravidity positively correlated with high fertilization in the Swabian Alb and with high mowing intensity in the Schorfheide. However, the number of eggs per gravid female in sexual species was not influenced by any land-use parameter. In parthenogens, numbers of eggs per gravid female increased in the Swabian Alb but decreased in the Schorfheide with increasing tree harvesting.

On the species level, land-use effects in forests were more pronounced than those in grasslands (Table 7, Online Appendix 6). In sexual species, about 6% were significant ‘losers’ to a high forest management index and about 4 and 7%, respectively, were ‘losers’ to tree harvesting and the proportion of non-native trees. A high proportion of dead wood with saw cuts had by far the greatest impact, resulting in 75% ‘losers’ among sexual species. Similarly, a high percentage of dead wood with saw cuts was correlated negatively with 87.5% of parthenogenetic species. ‘Winner’ species were more abundant in sexuals than in parthenogens, especially for the forest management index (about 12%) and the percentage of non-native trees (about 10%).

**Table 7:** Proportion of losers and winners species in sexual and parthenogenetic oribatid mite species in forests and grasslands

Forest	Formi		Idwcut		Iharv		Inonat	
	Loser	Winner	Loser	Winner	Loser	Winner	Loser	Winner
Sexual species	5.9	11.8	75	0	4.4	1.5	7.4	10.3
Parthenogenetic species	0	0	87.5	0	4.2	4.2	0	8.3

Grassland	LUI		Grazing		Mowing		Fertilization		
	Loser	Winner	Loser	Winner	Loser	Winner	Loser		Winner
Sexual species	1.9	7.4	0	0	0	3.7	0		1.9
Parthenogenetic species	0	0	0	6.7	0	0	0		0

*Formi* forest management index, *Idwcut* proportion of dead wood with saw cuts, *Iharv* proportion of trees harvested, *Inonat* proportion of non-native trees, *LUI* land-use index

In grasslands, we found only about 2% ‘losers’ in sexual species concerning the combined land-use index. On the other hand, about 7, 4 and 2% ‘winners’ were present in sexuals for the land-use index, mowing and fertilization, respectively. Parthenogenetic species did not react as ‘losers’ to any land-use parameter, but about 7% ‘winners’ were found on plots with high grazing intensity. On average, sexual and parthenogenetic oribatid mite species suffered equally from high land-use intensity in forests (23.3 vs. 22.9%) and grasslands (0.5 vs. 0%). On the other hand, ‘winner’ species, i.e., those species found on plots with high land-use intensity, were more common among sexual species in both forest (5.9 vs. 3.1%) and grassland (3.3 vs. 1.7%) habitats.

## Discussion

### Oribatid mite assemblages

In general, assemblages of oribatid mites in forests were more species-rich, had higher densities and were characterized by species with smaller body than those in grasslands. Furthermore, in both habitats and both regions sexually reproducing species and individuals dominated, except in Schorfheide forests, where the proportion of parthenogenetic individuals was higher than that of sexuals. Our findings are consistent with former studies (e.g., Maraun and Scheu 2000; Cianciolo and Norton 2006; Birkhofer et al. 2012; Erdmann et al. 2012; Wehner et al. 2016).

Although oribatid mites are present in almost all habitats around the world, their abundances vary with habitat and microhabitat conditions (e.g., Maraun and Scheu 2000; Wehner et al. 2016). As an important part of the decomposer system, oribatid mites are most numerous in temperate coniferous and deciduous forests, usually with highest abundances in soils with moder-humus (e.g., Moritz 1965; Scheu and Schulz 1996; Skubala 1999; Maraun and Scheu 2000; Lindo and Visser 2004; Kreibich and Alberti 2006). Former studies on oribatid mites in the framework of the Biodiversity Exploratories correlated their densities with forest type and age showing highest numbers in coniferous and lowest numbers in natural beech stands (Erdmann et al. 2012; Bluhm et al. 2016). Also in several grasslands, oribatid mites are among the most diverse and abundant arthropod groups (Behan-Pelletier and Kanashiro 2010).

Theory predicts that the distribution of the mode of reproduction in oribatid mites depends on the availability of resources in a certain habitat (Scheu and Drossel 2007). Higher percentages of parthenogenesis can be found in coniferous forests as compared to beech stands or acidic forests (Maraun et al. 2012, 2019; Bluhm et al. 2016) and even microhabitats within forests differ in the percentage of parthenogenetic inhabitants (Wehner et al. 2018). Although also in oribatid mites the majority of species reproduce by sexuality, the comparably high amount of parthenogenesis and the stable coexistence of sexually and parthenogenetically reproducing species remain remarkable.

Sex ratios within oribatid mite species were relatively constant among habitat and regions and seem to be species specific. In sexuals, for example, *O. calcarata* and *Chamobates cuspidatus* are usually characterised by relatively low percentages of females (below 50%), whereas the sex ratios of larger species such as *Eupelops* sp. and *Euzetes* sp. are highly female biased (Wehner et al. 2016, 2018; but see Luxton 1981). A female-biased sex-ratio may reduce the so-called twofold costs of sex which arise by the production of males: for example, in sexually reproducing onion thrips (*Thrips tabaci*) the population growth rate increases by reducing the frequency of male offspring and adjusts to the growth rate of parthenogenetic strains (Kobayashi and Hasegawa 2016). How far these findings can be transferred to coexisting sexual and parthenogenetic oribatid mite species needs further investigation of population dynamics.

In addition to their high percentages in oribatid mite assemblages, sexual species showed a higher proportion of gravidity and had 1.6× more eggs than parthenogens, which agrees with previous studies (Domes et al. 2007; Wehner et al. 2014). A higher gravidity rate and more eggs per gravid female may also reduce the twofold costs of sex by further adjusting the number of female progeny to that of parthenogens.

In parthenogenetically reproducing species of Enarthronota, Mixonomata and Nothrina, males were completely absent, but in *O. nova* and *Tectocepheus* spp. spanandric males were found. These males—which are assumed to be evolutionary artefacts (Taberly 1988)—are regularly found irrespective of habitat or season (Fujikawa 1988a, b; Wehner et al. 2014, 2016, 2018). If and how much these males contribute to the genetic diversity of parthenogenetic species is still unknown and needs further investigation.

### Influence of land-use intensity

Whereas effects of different land-use intensity on diversity and abundance of oribatid mite communities in forests were almost all negative, the strength and direction of effects in grasslands depended on region. On the species level, we found the same number of ‘losers’ in sexual and parthenogenetic species at high land-use intensities in both habitats, but ‘winner’ species were more common among sexuals.

In forests, land-use management can have strong local modulating effects influencing soil nutrient quality, water availability, the vegetation type of the shrub layer and tree composition (Zaitsev et al. 2013). In temperate forest soils, oribatid mites are an important component of the decomposer system, somehow buffering mechanisms against strong environmental changes and showing a multifaceted reaction to long-lasting disturbances (Zaitsev et al. 2002). The reaction of the soil community and especially oribatid mites to disturbance depends on the kind of land use (Marshall 2000; Birkhofer et al. 2012, 2017). Clear-cutting can reduce oribatid mite abundance up to 90% and changes oribatid mite community composition due to soil compaction and a following reduction and restructuring of the microbial community; partial-cut, stem-only harvesting or selective timber harvesting is usually less detrimental to forest floor microarthropods (Hope 2001; Battigelli et al. 2004; Bird et al. 2004; Lindo and Visser 2004). Furthermore, the removal of cut timber reduces important habitats (Déchêne and Buddle 2009). As several oribatid mite species are obligate members of the intra-log community (Siira-Pietikäinen et al. 2018; Skubala and Duras 2008), dead wood is important for diverse and unique oribatid mite assemblages (Déchêne and Buddle 2009).

In our study, the presence of unnatural dead wood—showing saw cuts from timber harvesting—had a negative impact on a high proportion of oribatid mite species in the litter, irrespective of their mode of reproduction. The presence of unnatural dead wood does not mean that the total amount of dead wood at a specific site is enhanced; it rather describes the enhanced proportion of dead wood with saw cuts relative to the total amount of dead wood. An explanation for the negative effect of unnatural dead wood on oribatid mite assemblages would require further experimental studies adding different kinds of dead wood, i.e., natural or artificial. For now, we assume that dead wood specialists may migrate from the litter to the available dead wood habitats, thereby lowering oribatid mite diversity and abundance in litter. However, this does not explain why natural dead wood would not have the same effect. It is also plausible that wood harvesting changed ecological conditions by, e.g., soil compaction due to timber removal, drought due to soil exposure after clear-cutting and other changes of the microclimate, and oribatid mite species may react by decreasing abundance. Probably the proportion of dead wood with saw cuts is the best indicator for overall harvesting disturbances for soil animals, reflecting all environmental changes mentioned before.

Furthermore, effects on animal communities are often difficult to separate (Minor et al. 2004; Wehner et al. 2018). A further parameter that can have an impact on soil animals by influencing soil structure, microclimate, water regime, microflora, and habitat diversity is the aboveground vegetation (Berg and Powluk 1984; Birkhofer et al. 2012). Birkhofer et al. (2012) showed that anthropogenic disturbances by different land-use types alter soil properties and strongly influence soil biota. In their study, oribatid mites were more abundant at sites with low pH and nitrate concentration.

In grasslands, land use often causes extremely perturbed soil by physical and/or chemical means. Fertilization or monocultures in grasslands lead to unnatural plant communities and pesticides and desiccation destroy habitat structure (Birkhofer et al. 2012; Lehmitz 2014). The alteration of physical soil properties reduces oribatid mite richness and density and alters the community structure. Furthermore, the usage of synthetic fertilizers has been shown to negatively affect abundance and diversity of oribatid mite communities by enhancing the nitrate concentration in soil (Birkhofer et al. 2012) which agrees to this study. Soil compaction due to livestock grazing and mowing can also decrease the abundance of small species and those intolerant to drought (Siepel 1996; Ivan 2009). In our study, grasslands showed far fewer ‘loser’ species than did forests, and up to 7.4% were ‘winners’ in sexuals. This indicates that grassland communities consist of more tolerant species that can cope with the greater amount of natural and human-introduced uncertainty in their habitat. Furthermore, oribatid mite species may profit from a stable soil microflora that gets continuous input of organic matter from litter and roots all year round (Vreeken-Buijs et al. 1998).

## Conclusion

In conclusion, not all land-use parameters showed negative effects on oribatid mite communities; in grasslands, also positive effects were observed. Biodiversity was generally more strongly affected than abundance. On the species level, sexual and parthenogenetic species suffered about equally from high land-use intensity, showing approximately equal proportions of ‘loser’ species. While most parthenogenetic species produced all-female offspring that may quickly recolonize disturbed habitats, sexual species hold up with female biased sex ratios, a higher percentage of gravidity and more eggs per gravid female which may enable those species to cope with land-use disturbances and to be ‘winners’ at high land-use intensities.

## Supplementary information

Below is the link to the electronic supplementary material.

Supplementary material 1 (XLSX 15 kb)

Supplementary material 2 (XLSX 15 kb)

Supplementary material 3 (XLSX 13 kb)

Supplementary material 4 (XLSX 12 kb)

Supplementary material 5 (XLSX 12 kb)

Supplementary material 6 (XLSX 24 kb)

## Data Availability

Data are deposited at the Bexis database ID 26446 and openly available under 10.25829/bexis.26446-3 or from the corresponding author upon request.

## Code availability

For statistical analyses, R version and RStudio version 3.5.2 were used. R scripts can be obtained from the corresponding author upon request.

## References

[CR1] Allan E, Manning P, Alt F (2015). Land use intensification alters ecosystem multifunctionality via loss of biodiversity and changes to functional composition. Ecol Lett.

[CR2] Attwood SJ, Maron M, House ARN, Zammit C (2008). Do arthropod assemblages display globally consistent responses to intensified agricultural land use and management?. Glob Ecol Biogeogr.

[CR3] Battigelli JP, Spence JR, Langor DW, Berch SM (2004). Short-term impact of forest soil compaction and organic matter removal on soil mesofauna density and oribatid mite diversity. Can J For Res.

[CR4] Behan-Pelletier VM (1999). Oribatid mite biodiversity in agroecosystems: role for bioindication. Agric Ecosyst Environ.

[CR5] Behan-Pelletier VM, Kanashiro D, Shorthouse JD, Floate KD (2010). Acari in grassland soils of Canada. Arthropods of Canadian grasslands (volume 1): ecology and interactions in grassland habitats.

[CR6] Bell G (1982). The masterpiece of nature. The Evolution and Genetics of Sexuality..

[CR100] Berg NW, Pawluk S (1984). Soil mesofaune studies under different vegetative regimes in Noth Central Alberta. Can J Soil Sci.

[CR7] Bird SB, Coulson RN, Fisher RF (2004). Changes in soil and litter arthropod abundance following tree harvesting and site preparation in a loblolly pine (*Pinus taeda* L.) plantation. For Ecol Manag.

[CR8] Birkhofer K, Bezemer TM, Bloem J (2008). Long-term organic farming fosters below and aboveground biota: implications for soil quality, biological control and productivity. Soil Biol Biochem.

[CR9] Birkhofer K, Schöning I, Alt F (2012). General relationships between abiotic soil properties and soil biota across spatial scales and different land-use types. PLoS ONE.

[CR10] Birkhofer K, Dietrich C, John K, Schorpp Q, Zaitsev AS, Wolters V (2016). Regional conditions and land-use alter the potential contribution of soil arthropods to ecosystem services in grasslands. Front Ecol Evol.

[CR11] Birkhofer K, Gossner MM, Diekötter T (2017). Land-use type and intensity differentially filter traits in above- and below-ground arthropod communities. J Anim Ecol.

[CR12] Bluhm C, Scheu S, Maraun M (2016). Temporal fluctuations in oribatid mites indicate that density-independent factors favour parthenogenetic reproduction. Exp Appl Acarol.

[CR13] Blüthgen N, Dormann CF, Prati D (2012). A quantitative index of land-use intensity in grasslands: integrating mowing, grazing and fertilization. Basic Appl Ecol.

[CR14] Chisté M, Mody K, Gossner MM, Simons NK, Köhler G, Weisser WW, Blüthgen N (2016). Losers, winners, and opportunists: how grassland land-use intensity affects orthopteran communities. Ecosphere.

[CR15] Chisté MN, Mody K, Kunz G, Gunczy J, Blüthgen N (2018). Intensive land use drives small-scale homogenization of plant- and leafhopper communities and promotes generalists. Oecologia.

[CR16] Cianciolo JM, Norton RA (2006). The ecological distribution of reproductive mode in oribatid mites, as related to biological complexity. Exp Appl Acarol.

[CR18] Culman SW, Young-Mathews A, Hollander AD, Ferris H, Sánchez-Moreno S, O`Green AT, Jackson LE (2010). Biodiversity is associated with indicators of soil ecosystem functions over a landscape gradient of agricultural intensification. Landscape Ecol.

[CR19] Déchêne AD, Buddle CM (2009). Decomposing logs increase oribatid mite assemblage diversity in mixedwood boreal forests. Biodivers Conserv.

[CR20] Domes K, Scheu S, Maraun M (2007). Resources and sex: soil re-colonization by sexual and parthenogenetic oribatid mites. Pedobiologia.

[CR21] Ehnes RB, Pollierer MM, Erdmann G, Klarner B, Eitzinger B, Digel C, Ott D, Maraun M, Scheu S, Brose U (2014). Lack of energetic equivalence in forest soil invertebrates. Ecology.

[CR22] Erdmann G, Scheu S, Maraun M (2012). Regional factors rather than forest type drive the community structure of soil living oribatid mites (Acari, Oribatida). Exp Appl Acarol.

[CR23] Fischer M, Bossdorf O, Gockel S (2010). Implementing large-scale and long-term functional biodiversity research: the Biodiversity Exploratories. Basic Appl Ecol..

[CR24] Fujikawa T (1988). Biology of *Tectocepheus velatus* (Michael) and *T. cuspidentatus* Knülle. Acarologia.

[CR25] Fujikawa T (1988). Biological features of *Oppiella nova* (Oudemans) in a nature farming field. Edaphologia.

[CR26] Gossner MM, Lewinsohn T, Kahl T (2016). Land-use intensification causes multitrophic homogenisation of grassland communities. Nature.

[CR27] Hope G (2001) The soil ecosystem of an ESSF forest and its response to a range of harvesting disturbances. Extension Note 53. B. C. Ministry of Forests Research Program, Victoria, B. C. Available from http://www.for.gov.bc.ac/hfd/pubs/Docs/En/En53.htm

[CR101] Ivan O (2009). Diversity and distribution of the oribatid mites (Acari, Oribatida) in some grassland ecosystems from the lower section of the prut meadow (Romania). Agranomie.

[CR28] Jost L (2006). Entropy and diversity. Oikos.

[CR29] Kahl T, Bauhus J (2014). An index of forest management intensity based on assessment of harvested tree volume, tree species composition and dead wood origin. Nat Conserv.

[CR30] Kempson D, Llyod M, Ghelardi R (1963). A new extractor for woodland litter. Pedobiologia.

[CR31] Klarner B, Ehnes RB, Erdmann G, Eitzinger B, Pollierer MM, Maraun M, Scheu S (2014). Trophic shift of soil animal species with forest type as indicated by stable isotope analysis. Oikos.

[CR32] Kobayashi K, Hasegawa E (2016). A female-biased sex ratio reduces the twofold cost of sex. Sci Rep.

[CR33] Kreibich E, Alberti G (2006). Reactions of oribatid mites (Acari: Oribatida) to changed forestry methods in the lowlands of northeastern Germany. Fragm Faun.

[CR34] Lehmitz R (2014). The oribatid mite community of a German peatland in 1987 and 2012—effects of anthropogenic desiccation and afforestation. Soil Org.

[CR35] Lehmitz L, Russell D, Hohberg K, Christian A, Xylander WER (2012). Active dispersal of oribatid mites into young soil. Appl Soil Ecol.

[CR36] Lehtonen J, Jennions MD, Kokko H (2012). The many costs of sex. Trends Ecol Evol.

[CR37] Liiri M, Häsä M, Haimi J, Setälä H (2012). History of land-use intensity can modify the relationship between functional complexity of the soil fauna and soil ecosystem services—a microcosm study. Appl Soil Ecol.

[CR38] Lindo Z, Visser S (2004). Forest floor microarthropod abundance and oribatid mite (Acari: Oribatida) composition following partial and clear-cut harvesting in the mixedwood boreal forest. Can J For Res.

[CR39] Luxton M (1981). Studies on the oribatid mites of a Danish beech wood soil. IV Developmental biology. Pedobiologia.

[CR40] Mangels J, Fiedler K, Schneider FD, Blüthgen N (2017). Diversity and trait composition of moths respond to land-use intensification in grasslands: generalists replace specialists. Biodivers Conserv.

[CR41] Maraun M, Scheu S (2000). The structure of oribatid mite communities (Acari, Oribatida): patterns, mechanisms and implications for future research. Ecography.

[CR42] Maraun M, Norton RA, Ehnes RB, Scheu S, Erdmann G (2012). Positive correlation between density and parthenogenetic reproduction in oribatid mites (Acari) supports the structured resource theory of sexual reproduction. Evol Ecol Res.

[CR102] Maraun M, Caruso T, Hense J (2019). Parthenogenetic vs. sexual reproduction in oribatid mite communities. Ecol Evol.

[CR43] Marshall VG (2000). Impacts of forest harvesting on biological processes in northern forest soils. For Ecol Manag.

[CR103] Maynard Smith J (1978). The evolution of sex.

[CR104] Minor M, Volk TA, Norton RA (2004). Effects of site preparation techniques on communities of soil mites (Acari: Oribatida, Acari: Gamasida) under short-rotation forestry plantings in New York, USA. Appl Soil Ecol.

[CR44] Moritz M (1965). Untersuchungen über den Einflus von Kahlschlagmaßnahmen auf die Zusammensetzung von Hornmilbengemeinschaften (Acari: Oribatai) norddeutscher Laub- und Kiefernmischwälder. Pedobiologia.

[CR105] Newbold T, Hudson LN, Hill SLL (2005). Global effects of land use on local terrestrial biodiversity. Nature.

[CR45] Norton RA, Dindal D (1990). Acarina: Oribatida. Soil biology guide.

[CR47] Norton RA, Palmer SC, Schuster R, Murphy PW (1991). The distribution, mechanisms, and evolutionary significance of parthenogenesis in oribatid mites. The Acari: reproduction, development and life-history strategies.

[CR48] Oksanen J, Blanchet FG, Friendly M et al. (2019). vegan: community Ecology Package. R package version 2.5-6. https://CRAN.R-project.org/package=vegan

[CR49] Penone C, Allan E, Soliveres S (2019). Specialisation and diversity of multiple trophic groups are promoted by different forest features. Ecol Lett.

[CR50] R Core Team (2018) R: a language and environment for statistical computing. Vienna: R Foundation for Statistical Computing. Available at https://www.R-project.org/

[CR52] Schatz H, Behan-Pelletier V (2008). Global diversity of oribatids (Oribatida: Acari: Arachnida). Hydrobiologia.

[CR53] Schatz H, Behan-Pelletier VM, OConnor BM, Norton RA (2011) Suborder Oribatida van der Hammen, 1968. In: Zhang Z-Q (ed) Animal biodiversity: an outline of higher level classification and survey of taxonomic richness. Zootaxa 3148:141–148 10.11646/zootaxa.3703.1.1 26146682

[CR54] Scheu S, Drossel B (2007). Sexual reproduction prevails in a world of structured resources in short supply. Proc R Soc B.

[CR55] Scheu S, Schulz E (1996). Secondary succession, soil formation and development of a diverse community of oribatids and saprophagous soil macro-invertebrates. Biodivers Conserv.

[CR56] Seastedt TR (1984). The role of microarthropods in decomposition and mineralization processes. Annu Rev Entomol.

[CR57] Seastedt TR, Crossley DA (1981). Microarthropod response following cable logging and clear-cutting in the southern Appalachians. Ecology.

[CR106] Siepel H (1996). The importance of unpredictable and short-term environmental extremes for biodiversity in oribatid mites. Biodiv Letters.

[CR107] Siira-Pietikäinen A, Penttinen R, Huhta V (2018). Oribatid mites (Acari: Oribatida) in boreal forest floor and decaying wood. Pedo.

[CR59] Skubala P, Bruin J, van der Geest LPS, Sabelis MW (1999). Comparison of adult oribatid mites (Acari, Oribatida) from three mountain forests in Poland: I. Abundance, biomass and species richness. Ecology and evolution of Acari.

[CR60] Skubala P, Duras M (2008). Do decaying logs represent habitat islands? Oribatid mite communities in dead wood. Anal Zool.

[CR61] Taberly G (1988). Recherches sur la parthénogenèse thélythoque de deux espèces d’acariens oribatides: *Trhypochthonius tectorum* (Berlese) et *Platynothrus peltifer* (Koch). IV Observations sur les mâles ataviques. Acarologia.

[CR108] Vreeken-Buijs MJ, Hassink J, Brussaard L (1998). Relationship of soil microarthropod biomass with organic matter and pore size distribution in soils under different land use. Soil Biol Biochem.

[CR62] Wallwork JA (1983). Oribatids in forest ecosystems. Annu Rev Entomol.

[CR63] Wehner K, Scheu S, Maraun M (2014). Resource availability as driving factor of the reproductive mode in soil microarthropods (Acari, Oribatida). PLoS ONE.

[CR64] Wehner K, Norton RA, Blüthgen N, Heethoff M (2016). Specialization of oribatid mites to forest microhabitats—the enigmatic role of litter. Ecosphere.

[CR65] Wehner K, Heethoff M, Brückner A (2018). Sex ratios of oribatid mite assemblages differ among microhabitats. Soil Org.

[CR66] Weigmann G (2006) Hornmilben (Oribatida). In: Dahl (ed) Tierwelt Deutschlands 76. Goecke and Evers, Keltern

[CR67] Wolters V (2001). Biodiversity of soil animals and its function. Eur J Soil Biol.

[CR68] Zaitsev AS, Chauvat M, Pflug A, Wolters V (2002). Oribatid mite diversity and community dynamics in a spruce chronosequence. Soil Biol Biochem.

[CR69] Zaitsev AS, van Straalen NM, Berg MP (2013). Landscape geological age explains large scale spatial trends in oribatid mite diversity. Landscape Ecol.

